# A gene stacking approach leads to engineered plants with highly increased galactan levels in Arabidopsis

**DOI:** 10.1186/s12870-014-0344-x

**Published:** 2014-12-10

**Authors:** Vibe M Gondolf, Rhea Stoppel, Berit Ebert, Carsten Rautengarten, April JM Liwanag, Dominique Loqué, Henrik V Scheller

**Affiliations:** Feedstocks Division, Joint BioEnergy Institute, Emeryville, California 94608 USA; Physical Biosciences Division, Lawrence Berkeley National Laboratory, Berkeley, California 94720 USA; Department of Plant and Environmental Sciences, University of Copenhagen, DK-1871 Frederiksberg C, Denmark; Department of Plant and Microbial Biology, University of California, Berkeley, California 94720 USA

**Keywords:** Plant cell wall, UDP-glucose 4-epimerase, Galactan, Pectin, Arabidopsis, *Populus*, Gene stacking, GalS1, NST1, Artificial positive feedback loop

## Abstract

**Background:**

Engineering of plants with a composition of lignocellulosic biomass that is more suitable for downstream processing is of high interest for next-generation biofuel production. Lignocellulosic biomass contains a high proportion of pentose residues, which are more difficult to convert into fuels than hexoses. Therefore, increasing the hexose/pentose ratio in biomass is one approach for biomass improvement. A genetic engineering approach was used to investigate whether the amount of pectic galactan can be specifically increased in cell walls of Arabidopsis fiber cells, which in turn could provide a potential source of readily fermentable galactose.

**Results:**

First it was tested if overexpression of various plant UDP-glucose 4-epimerases (UGEs) could increase the availability of UDP-galactose and thereby increase the biosynthesis of galactan. Constitutive and tissue-specific expression of a poplar UGE and three Arabidopsis UGEs in Arabidopsis plants could not significantly increase the amount of cell wall bound galactose. We then investigated co-overexpression of *At*UGE2 together with the β-1,4-galactan synthase GalS1. Co-overexpression of *At*UGE2 and GalS1 led to over 80% increase in cell wall galactose levels in Arabidopsis stems, providing evidence that these proteins work synergistically. Furthermore, *At*UGE2 and GalS1 overexpression in combination with overexpression of the NST1 master regulator for secondary cell wall biosynthesis resulted in increased thickness of fiber cell walls in addition to the high cell wall galactose levels. Immunofluorescence microscopy confirmed that the increased galactose was present as β-1,4-galactan in secondary cell walls.

**Conclusions:**

This approach clearly indicates that simultaneous overexpression of *At*UGE2 and GalS1 increases the cell wall galactose to much higher levels than can be achieved by overexpressing either one of these proteins alone. Moreover, the increased galactan content in fiber cells while improving the biomass composition had no impact on plant growth and development and hence on the overall biomass amount. Thus, we could show that the gene stacking approach described here is a promising method to engineer advanced feedstocks for biofuel production.

## Background

Plant cell walls are complex structures composed of polysaccharides influencing plant morphology, defense, growth, and signaling. They also constitute the most abundant biomaterial on earth and have the potential to provide a source of cheap sugars for industrial biotechnology. In lignocellulosic biomass, the cell wall polysaccharides comprise mostly cellulose and glucuronoxylan, a hemicellulose, embedded in highly cross-linked lignin polymers, which protect the polysaccharides from chemical and enzymatic degradation. The hemicellulosic fraction is mostly composed of pentoses (such as xylose and arabinose), which unlike hexoses cannot be easily fermented by yeast into fuels. Two main goals of engineering plants with an altered cell wall composition in order to lower costs and improve efficiency of biofuel production is to decrease recalcitrance by decreasing the lignin content or altering the lignin composition [1,2] or to reduce the content of glucuronoxylan and at the same time increasing the content of polysaccharides composed of a larger proportion of fermentable hexoses [3].

β-1,4-galactan is found as sidechains attached to rhamnogalacturonan I and is generally not highly abundant in lignocellulosic biomass. However, since β-1,4-galactan is composed entirely of galactose residues, which can be easily fermented by yeast, an increased content of this polysaccharide would potentially improve the biomass composition for biofuel purposes. In this study we used a genetic engineering approach to specifically increase the amount of β-1,4-galactan in stem cell walls.

Cell wall polysaccharides are synthesized by glycosyltransferases, which catalyze the formation of glycosidic linkages to form glycosides. During this process monosaccharides from activated sugar substrates are transferred onto glycosyl acceptors. Donor sugars are usually nucleotide sugars, while acceptors can be oligo- or polysaccharides, lipids, proteins, nucleic acids or other small molecules [4]. Recently, the Arabidopsis glycosyltransferase GALACTAN SYNTHASE 1 (GalS1) was shown to be a β-1,4-Galactan synthase*.* Constitutive overexpression of GalS1 in Arabidopsis wild-type plants led to a 50% increase in cell wall bound galactose in leaves [5]. Loss-of-function mutants in *Gals1* or its homologs *Gals2* and *Gals3* had a larger decrease in cell wall bound galactose in leaves than in stems [5], which suggested that the supply of UDP-galactose might be limiting in stems.

Nucleotide sugars are synthesized by different types of interconverting enzymes such as epimerases, decarboxylases and dehydrogenases. Most of these enzymes are located in the cytosol but some are found within the Golgi lumen [6]. Changes in nucleotide sugar pools can affect the biosynthesis of cell wall polysaccharides, as shown for example for the UDP-glucose dehydrogenase (UGD) double mutant *ugd2/ugd3,* which exhibits significantly reduced cell wall arabinose, xylose, apiose, and galacturonic acid levels [7]. Similarly, the UDP-xylose 4-epimerase (UXE) mutant *mur4* has a 50% decrease in cell wall arabinose [8].

The nucleotide sugar UDP-galactose is formed from UDP-glucose by UDP-glucose 4-epimerase (UGE). Five UGE isoenzymes exist in Arabidopsis (*At*UGEs), all of which have been functionally characterized *in vivo* [9-11]. Differences in the expression pattern, kinetics and amino acid sequences of the five *At*UGEs suggest that these isoenzymes have an overlapping, but not identical function in plants. UGE-overexpressing plants and knockout mutants have been generated, but specific roles for each of the UGEs could not be unambiguously concluded from those experiments. Only the *AtUGE4* knockout mutant ROOT HAIR DEFICIENT 1 *(rhd1/UGE4*^*rhd1*^*)* produces a visible phenotype. Roots of *uge4* mutants are shorter as compared to the wild type, and the root epidermis cells are swollen due to a defective synthesis of xyloglucan and type II arabinogalactan [10,12,13]. All five *At*UGEs can rescue this phenotype when expressed under the control of the constitutive cauliflower mosaic virus 35S promoter [9]. In double, triple and quadruple *At*UGE mutants, Rösti et al. [10] observed growth defects and cell wall compositional changes that suggested a partial functional overlap of the five UGE isoenzymes. The authors concluded that *At*UGE2 and *At*UGE4 affect vegetative growth and cell wall carbohydrate biosynthesis whereas *At*UGE1 and *At*UGE5 act in stress situations, and *At*UGE3 seems to be important for pollen development. Analysis of global co-expression profiles led to the conclusion that *At*UGE1 and *At*UGE3 are co-expressed with putative trehalose-6-phosphate synthase genes, whereas *At*UGE2, −4, and −5 are co-expressed with various known glycosyltransferases and other cell wall biosynthetic enzymes, suggesting that *At*UGE1 and *At*UGE3 might preferentially act in the UDP-galactose (UDP-Gal) to UDP-glucose (UDP-Glc) direction, while *At*UGE2, −4, and −5 might act in the UDP-Glc to UDP-Gal direction *in vivo* [9].

**Figure 1:**
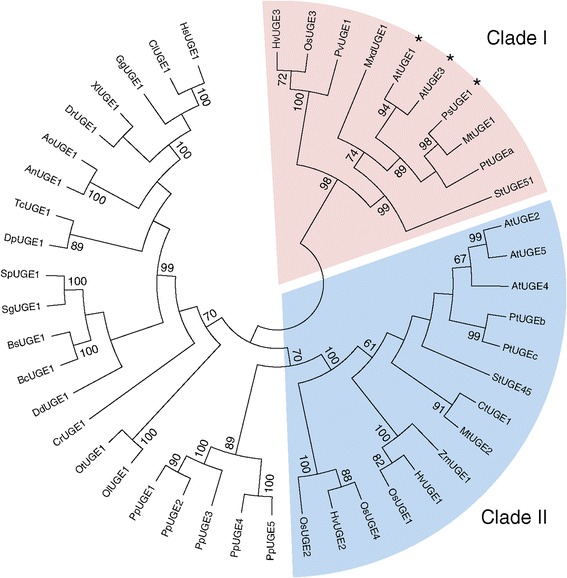
**Cladogram of UDP-glucose 4-epimerases.** The phylogenetic relationships of UGEs from different organisms are shown. Two vascular plant UGE families described by Kotake et al. [11] are highlighted. UGEs that have documented UDP-Xyl 4-epimerase activity are marked with asterisks. The tree was generated using the Neighbor-Joining method, the bootstrap consensus tree inferred from 1000 replicates (MEGA6 program). Bootstrap values greater than 60% are indicated. Species and accession numbers are described in the Methods section.

All UGE isoenzymes can interconvert UDP-Glc and UDP-Gal *in vitro*, although they show differences in the substrate affinity and reaction requirements [9,11]. Interestingly, two isoenzymes, *At*UGE1 and *At*UGE3 have been shown to also interconvert UDP-xylose (UDP-Xyl) and UDP-arabinose (UDP-Ara) [11]. This bifunctionality is reflected in the amino acid sequence of the different *At*UGEs. Phylogenetic analysis of UGE homologs in different organisms revealed that UGEs in plants are distributed in two plant-specific clades with *At*UGE1 and *At*UGE3 grouping together in clade I, while *At*UGE2, −4, and −5 group together in clade II (Figure 1). Besides *At*UGE1 and *At*UGE3, the pea *Ps*UGE1 has also been shown to interconvert UDP-Xyl and UDP-Ara [11]. *Ps*UGE1 is also located in UGE clade I, indicating that this clade contains additional bifunctional UGEs. Overexpression of two potato UGEs (*St*UGE45 and *St*UGE51) led to an increase of galactose in potato tuber cell walls [14] consistent with the hypothesis that the amount of available UDP-Gal rather than that of galactosyltransferases can be the limiting factor for the accumulation of cell wall galactose.

In the present study, we overexpressed different UGEs from Arabidopsis and poplar, in order to increase the level of cell wall galactose and thereby improve the C_6_/C_5_ sugar ratio. In addition to constitutive overexpression of one poplar and three Arabidopsis UGEs, we expressed one of the Arabidopsis UGEs (*At*UGE2) under the control of the secondary cell wall specific promoter pIRX5 together with the master transcription factor NST1. IRX5 is one of the catalytic subunits of the cellulose synthase complex in secondary cell walls [15] and its expression is induced by the transcription factor NST1 [16]. Expression of NST1 under the pIRX5 promoter has been shown to create a transcriptional positive feedback loop enhancing overall expression of secondary cell wall biosynthesis genes and thereby increasing secondary cell wall deposition in fiber cells [2]. Finally, constitutive and fiber-specific promoter *At*UGE2 constructs were expressed in the background of transgenic plants overexpressing the galactan synthase GalS1 [5].

While expression of any of the four UGEs alone did not alter the galactose content significantly, regardless of the promoter used, co-overexpression of *At*UGE2 and GalS1 led to an increase in the cell wall galactose content of stems of up to 80%.

## Results and discussion

### *Populus trichocarpa* UGEc is bifunctional *in vitro*

**Figure 2:**
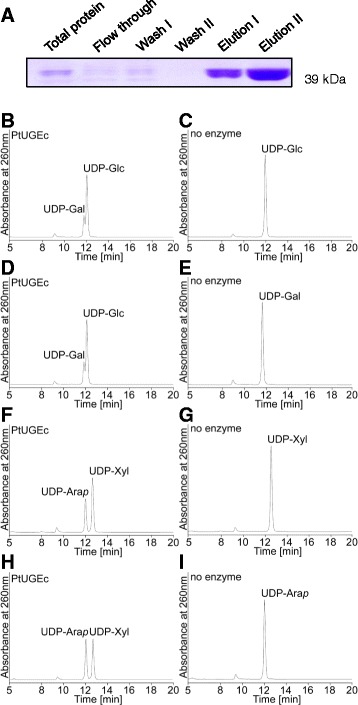
**UDP-glucose 4-epimerase activity of recombinant** ***Pt*** **UGEc protein. (A)** SDS-PAGE analysis of different fractions from the purification of His-*Pt*UGEc. **(B-I)** High-performance liquid chromatography (HPLC) chromatograms of nucleotide sugars incubated for 30 min with (left panels) or without (right panels) recombinant *Pt*UGEc protein.

In order to compare the performance of different UGEs for the engineering purpose, we first cloned a UGE from poplar since it might be preferable to use a poplar gene for the ultimate translation on the engineering approach to a biofuel crop such as poplar. Out of the UGEs encoded in the *Populus trichocarpa* genome, we selected one, referred to as *Pt*UGEc (XP_002299469, POPTR_0001s10700g). Due to its higher sequence similarity to the non-bifunctional *At*UGEs of Clade II that preferentially act in interconverting UDP-Glc to UDP-Gal (Figure 1), *Pt*UGEc seemed a good candidate for generating higher amounts of galactan. The *in vitro* activity of purified His-*Pt*UGEc (Figure 2A) was tested in reactions with different nucleotide sugars (UDP-Glc, UDP-Gal, UDP-Xyl, UDP-Ara). Proportions of UDP-sugars after incubation for 30 min show that the *Pt*UGEc enzyme is bifunctional, interconverting UDP-Glc and UDP-Gal, as well as UDP-Xyl and UDP-Ara (Figure 2B). The enzymatic reaction does not require the addition of NAD^+^, most likely because of non-covalent binding and co-purification of NAD^+^ together with the enzyme, as it has been shown for the barley *Hv*UGE1 [17]. Final UDP-Gal and UDP-Glc concentrations were the same in reactions starting with UDP-Gal or with UDP-Glc, indicating that equilibrium levels were reached. The reactions starting with either UDP-Xyl or UDP-Ara did not completely reach the equilibrium level, suggesting that the reaction rates for UDP-Xyl/UDP-Ara interconversion are slower than for UDP-Gal/UDP-Glc. The bifunctional enzymatic characteristics of *Pt*UGEc were somewhat unexpected for a UGE belonging to Clade II since other UGEs from this clade have been found to be specific for UDP-Glc/UDP-Gal interconversion. However, structural features that determine bifunctionality vs. mono-functionality are not known yet.

### All UGE constructs complement the *UGE4*^*rhd1*^ phenotype

**Figure 3:**
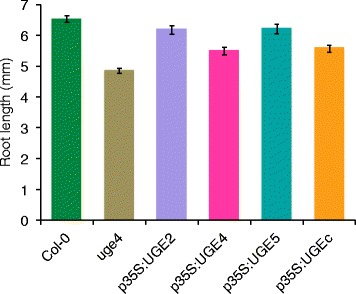
**Overexpression of** ***At*** **UGE2,** ***At*** **UGE4,** ***At*** **UGE5, or** ***Pt*** **UGEc rescues the** ***uge4*** **mutant phenotype.** The UGE expressing plants rescue the *uge4* phenotype to various extent. Average root length of Col-0, *uge4* and transgenic seedlings in *uge4* background was determined. Data show mean ± SD (n = 50).

In order to verify the functionality of UGE constructs, *Pt*UGEc and the Arabidopsis *At*UGE2, *At*UGE4, and *At*UGE5 proteins were overexpressed under the control of the constitutive cauliflower mosaic virus 35S promoter in the Arabidopsis *uge4* mutant background. Loss of function of *UGE4* results in a reduced root elongation rate and swelling of root epidermal cells probably as a result of defective cell wall matrix carbohydrate biosynthesis [12,18]. Thus, a simple visual screen can confirm complementation of the wild-type phenotype and thereby not only expression but also functionality of the UGE proteins. The four different UGE constructs all suppressed the root epidermal swelling and the reduced root length confirming previous UGE complementation results published by Barber et al. [9] and demonstrating that the poplar *Pt*UGEc is functional *in planta* (Figure 3). The slight root length decrease in our UGE overexpressor plants as compared to wild type indicates however, that complementation is not complete. Expression of *At*UGE2 and *At*UGE5 resulted in almost complete complementation.

### Plants overexpressing UGE show no increase in cell wall bound galactose in leaf or stem cell walls

**Figure 4:**
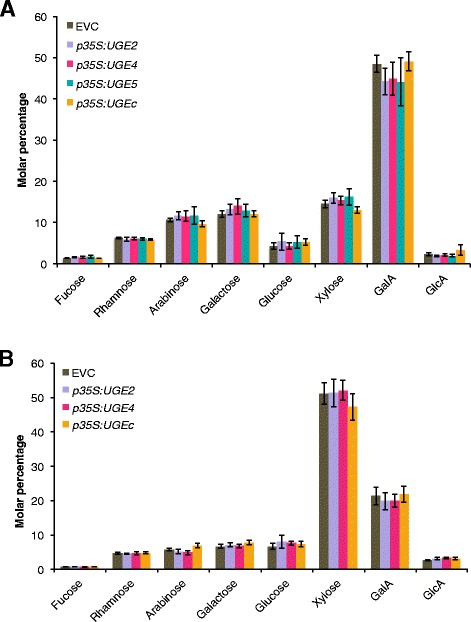
**Cell wall monosaccharide composition analysis of leaf (A) and stem (B) material from UGE overexpression plants.** Monosaccharide composition analysis of leaf **(A)** and stem **(B)** cell walls of plants constitutively overexpressing *At*UGE2, *At*UGE4, *At*UGE5 did not show any significant differences compared to the empty vector control (EVC) (p > 0.05, pairwise t-test with Holm-Bonferroni correction for family-wise error rate). Monosaccharide levels are shown as molar percentage ± SD (n = 6). GalA, α-d-galacturonic acid; GlcA, α-d-glucuronic acid.

All *At*UGE2, *At*UGE4, *At*UGE5, or *Pt*UGEc overexpressing Arabidopsis plants had a growth phenotype similar to wild-type Col-0 and the empty vector control plants. The monosaccharide composition of non-cellulosic polysaccharides from leaves and stems was analyzed by high-performance anion exchange chromatography with pulsed amperometric detection (HPAEC-PAD). Two to four independent transgenic lines were analyzed for each construct in the T2 generation. Data for one representative line for each construct is shown in Figure 4. The transgenic Arabidopsis UGE lines had no significant changes in sugar composition compared to the empty vector control plants in leaf cell walls (Figure 4A) or in stem cell walls (Figure 4B).

Recently, we have shown that overexpression of GalS1 *in planta* can lead to a significant 40% increase in total cell wall galactose in leaves [5]. Thus, the UDP-Gal substrate does not seem to be limiting for galactose incorporation into the cell wall in leaves and therefore an increase in interconverting UGE enzymes is not necessarily expected to lead to an increase in cell wall galactan.

### Cell wall bound galactose levels are increased in stem cell walls of co-overexpressers

**Figure 5:**
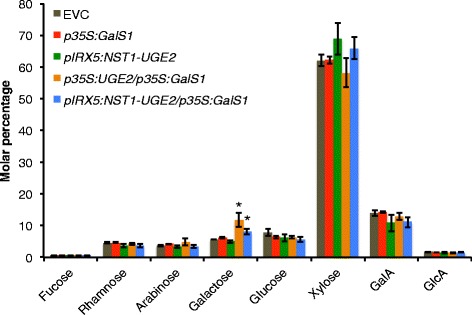
**Cell wall monosaccharide composition analysis of stems from plants co-overexpressing** ***At*** **UGE2 and GalS1.** The monosaccharide composition of stems from plants in the T3 generation expressing either *p35S:GalS1* or *pIRX5:NST1-UGE2* or co-overexpressing either *pIRX5:NST1-UGE2/p35S:GalS1* or *p35S:UGE2*/*p35S:GalS1* was determined. Monosaccharide levels are shown as molar percentage ± SD (n = 5). Significantly increased levels of galactose (p ≤ 0.01, indicated with asterisks) were found with the two co-expressing constructs *pIRX5:NST1-UGE2/p35S:GalS1* and *p35S:UGE2*/*p35S:GalS1*, while no other sugars were different from empty vector control plants (EVC) in any of the transgenic plants (p > 0.05). Analysis in the T2 generation of three independent lines for each construct showed the same results. The data were analyzed by pairwise t-test with Holm-Bonferroni correction for family-wise error rate. GalA, α-d-galacturonic acid; GlcA, α-d-glucuronic acid.

Since overexpression of *At*UGE2, *At*UGE4, *At*UGE5, or *Pt*UGEc alone did not result in a significant increase of total galactose in stems, we designed a gene stacking approach by co-expressing *At*UGE2 together with the galactan synthase GalS1. AtUGE2 was chosen because it showed efficient complementation of the *uge4* root phenotype (Figure 3). Although we had initially preferred to use a poplar UGE, PtUGEc was not a good choice because of its bispecificity and incomplete ability to complement *uge4*. We designed two different constructs for *At*UGE2 expression. One under the control of the constitutive 35S promoter (*p35S:UGE2*) and a second fiber-specific construct (*pIRX5:NST1-UGE2*). The fiber-specific construct is under control of the *IRX5* promoter and in addition expresses the transcription factor NST1 leading to a positive-artificial feedback loop and increased wall thickness in fiber cells, as previously reported [2]. In this construct *UGE2* is expressed from the same *pIRX5* promoter, separated from *NST1* with the 2A sequence from foot-and-mouth disease virus allowing coordinate expression of multiple proteins [19]. The stem cell wall composition was analyzed in the T2 generation for three independent lines for each construct. For each construct there was no difference between the independent lines and one line was selected for confirmation of the results in the T3 generation (Figure 5). Expression of *p35S:UGE2* and *pIRX5:NST1-UGE2*, respectively, in the background of plants constitutively overexpressing GalS1 (*p35S:GalS1*) led to significantly increased galactose levels (p ≤ 0.01) as shown by analysis of the monosaccharide composition of cell walls from stems (Figure 5). While *p35S:UGE2/p35S:GalS1* plants showed a galactose increase of more than 80%, the *pIRX5:NST1-UGE2/p35S:GalS1* galactose levels were only increased by 44% as compared to empty vector control (EVC) plants (Figure 5). Plants expressing *p35S:GalS1* alone had only a slight increase in stem wall bound galactose, and no galactose increase was observed when the *pIRX5:NST1-UGE2* construct was incorporated in the wild-type background. The apparent increase in xylose in plants containing the *pIRX5:NST1-UGE2* construct for overexpression of the feedback-loop construct with NST1 could be expected in plants with increased fiber cell wall density and more xylan. However, the xylose content in these plants is not significantly different from the control (p > 0.05 even without Holm-Bonferroni correction). These results show that GalS1 is limiting for galactan synthesis in both leaves and stems while UGEs are not. However, when GalS1 is overexpressed, *At*UGE2 or other UGEs seem to become limiting for how much galactan can be accumulated in cell walls of Arabidopsis plant stems. The 80% increase in cell wall galactose may not be the limit for what can be achieved. UDP-Gal formed by UGE2 must be transported into the Golgi lumen to be used by GalS1, and it is possible that the transport becomes limiting when both UGE2 and GalS1 are overexpressed. Recently, a UDP-Gal transporter URGT1 has been characterized, the overexpression of which results in increased cell wall galactan in leaves [20]. We are currently investigating the effect of overexpressing the transporter together with UGE2 and GalS1.

**Figure 6:**
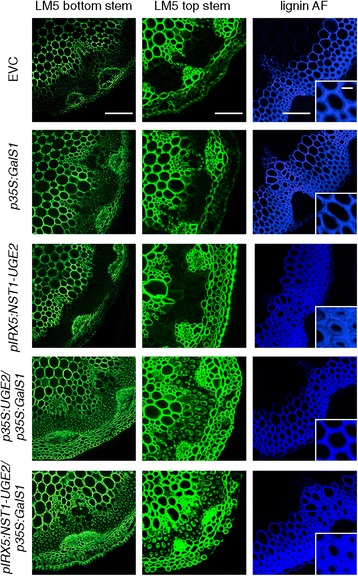
**Galactan and lignin detection in stem sections.** β-1,4-galactan was detected by immunofluorescence microscopy using the LM5 antibody. Stem sections from the top and bottom of inflorescence stems were analyzed. Plants co-expressing either *pIRX5:NST1-UGE2/ p35S:GalS1* or *p35S:UGE2*/*p35S:GalS1* show a very strong labeling of galactan in the secondary wall as compared to the empty vector control or plants expressing only *p35S:GalS1* or *pIRX5:NST1-UGE2*. Visualization of lignin autofluorescence using a confocal microscope under UV light shows the increase in fiber cell wall density with constructs using the *pIRX5* promoter. Bars are 100 μm for bottom stem and lignin autofluorescence pictures, 50 μm for top stems and 10 μm for lignin close ups.

The increase in galactose of *p35S:UGE2/p35S:GalS1* and *pIRX5:NST1-UGE2/p35S:GalS1* co-overexpressor plants was further investigated by immunofluorescence microscopy of stem sections (Figure 6). Although immunofluorescence microscopy is not easily quantified, the detection of the LM5 galactan epitope was strongly increased specifically in the secondary cell walls of top and bottom stem sections of *p35S:UGE2/p35S:GalS1* and *pIRX5:NST1-UGE2/p35S:GalS1* co-overexpressors, as compared to overexpressor lines of *p35S:GalS1* or *pIRX5:NST1-UGE2* alone, or the empty vector control. The fiber-specific pIRX5 constructs in addition resulted in highly thickened cell walls as an effect of overexpressing NST1 under the IRX5 promoter and as visualized by lignin autofluorescence using a confocal microscope (Figure 6). On the one hand this increase in biomass density is highly desirable to improve the cost-effectiveness of lignocellulosic bioenergy production. On the other hand however, the increase in xylan rather results in more recalcitrance and is counteracting the increase in the C6/C5 sugar ratio, which is obtained due to increased galactan deposition. Therefore, future implementation of galactan overexpression could be improved by simultaneously downregulating xylan deposition in fiber cells. This could be achieved by using mutants deficient in xylan that have been complemented by reintroducing xylan biosynthesis specifically into the xylem vessels in order to restore wild type-like growth of the plants, as recently described [3]. Conceivably, the lignin content in fiber cells also needs to be decreased.

## Conclusions

Rescuing of the *uge4* root phenotype proved functionality of the constitutively expressed poplar *p35S:UGEc* and the three Arabidopsis *p35S:UGE2*, *p35S:UGE4* and *p35S:UGE5* constructs. Overexpression of any of the four UGE proteins alone did not increase the total cell wall galactose content in Arabidopsis leaves or stems. However, our gene stacking approach, combining overexpression of *AtUGE2* and *GalS1*, clearly showed that it is possible to engineer plants with an even higher galactose content than *GalS1* overexpressing plants by combining the overexpression of multiple genes into one plant. The Arabidopsis plants obtained have a more than 80% increase in stem galactose levels as compared to wild-type or empty vector control plants. Importantly, these transgenic plants exhibit no impairment of growth and development. Our study shows the promise of the gene stacking approach for engineering plants with improved properties for biofuel applications.

## Methods

### Phylogenetic analysis

Phylogenetic analyses were conducted in MEGA6 [21]. The bootstrap consensus tree inferred from 1000 replicates was taken to represent the evolutionary history of the taxa analyzed using the Neighbor-Joining method. The percentages of replicate trees in which the associated taxa clustered together in the bootstrap test are shown next to the branches retaining only groups with a frequency ≥60%. Species and Genbank accession numbers are: An, *Aspergillus niger* [XP_001401007]; At, *Arabidopsis thaliana* [AEE28928, UGE1; AEE84827, UGE2; AEE34065, UGE3; AEE34241, UGE4; AEE82951, UGE5]; Ao, *Aspergillus oryzae* [XP_001827449]; Bc, *Bacillus cereus* [ZP_01180393]; Bs, *Bacillus subtilis* [P55180]; Cl, *Canis lupus* [XP_544499]; Cr, *Chlamydomonas reinhardtii* [XP_001698706]; Ct, *Cyamosis tetragonoloba* [O65781]; Dd, *Dictyostelium discoideum* [XP_643834]; Dp, *Drosohila pseudoobscura* [XP_001352806]; Dr, *Danio rerio* [NP_001035389]; Gg, *Gallus gallus* [XP_417833]; Hs, *Homo sapiens* [Q14376]; Hv, *Hordeum vulgare* [AAX49504, UGE1; AAX49505, UGE2; AAX49503, UGE3]; Mt, *Medicago truncatula* [ACJ85116, UGE1; ACJ84690, UGE2]; Mxd, *Malus x domestica* [BAF51705]; Ol, *Ostreococcus lucimarinus* [XP_001419325]; Os, *Oryza sativa* [BAF18426, UGE1; BAF23582, UGE2; BAF25641, UGE3; BAF24783, UGE4]; Ot, *Ostreococcus tauri* [CAL54894]; Pp, *Physcomitrella patens* [XP_001768301, UGE1; XP_001777464, UGE2; XP_001775163, UGE3; XP_001751529, UGE4; XP_001771084, UGE5]; Ps, *Pisum sativum* [AB381885]; Pt, *Populus trichocarpa* [XP_002304478, UGEa: XP_002303653, UGEb; XP_002299469, UGEc;] Pv, *Paspalum vaginatum* [BAE92559]; Sg, *Streptococcus gordonii* [AAN64559]; St, *Solanum tuberosum* [AAP42567, UGE45; AAP97493, UGE51]; Sp, *Streptococcus pneumonia* [ZP_01825231]; Tc, *Tribolium castaneum* [XP_968616]; Xl, *Xenopus laevis* [NP_001080902]; Zm, *Zea mays* [AAP68981].

### Plant material and vectors

**Figure 7:**
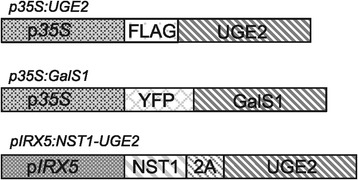
**Schematic presentation of the gene constructs used.** The constructs used to generate the transgenic plants used for Figures 4, 5 and 6 incorporated either the *p35S* or the Arabidopsis *pIRX5* promoter. The UGE2 and GalS1 proteins were expressed with tags as indicated in the two upper constructs. The *pIRX5:NST1-UGE2* construct had the two open reading frames separated by a 2A sequence. For additional details see Methods and Table 2.

All *Arabidopsis thaliana* (L.) Heynh. wild-type and mutant plant lines used were of ecotype Columbia-0 (Col-0). The *At*UGE4 mutant *uge4*/*rhd1-1* (At1g64440, CS2257) was obtained from the Arabidopsis Biological Resource Center (ABRC, http://www.arabidopsis.org). Plants overexpressing YFP-GalS1 (*p35S:GalS1*) have been previously described [5]. *Populus trichocarpa* Nisqually-1 leaf tissue was kindly donated by Dr. Lee Gunter (Oak Ridge National Laboratory). Entry vectors containing *AtUGE2*, *AtUGE4* and *AtUGE5* cDNA (At4g23920, At1g64440, At4g10960) and the Gateway-compatible plant transformation vectors pMDC32 and pMDC43 were obtained from ABRC. The destination vector pTKan-pIRX5-GWR3R2 was made as described [1] except that it had attR2 and attR3 recombination sites. Gene constructs used to generate transgenic plants are shown in Figure 7.

### Generation of vectors and transformation of plants

Flag-tagged UGE constructs were generated by PCR using Phusion Polymerase (Thermo Scientific) with primers fU2-F, fU2-R (*At*UGE2); fU4-F, fU4-R (*AtUGE4*); fU5-F, fU5-R (*At*UGE5); and fUc-F, fUc-R (*Pt*UGEc) (Table 1), and cloned into vectors pMDC32 and pMDC43 using Gateway technology (Life Technologies). The fiber expressed *At*UGE2 construct was generated in 2 steps. The sequence encoding for the NST1-2A-AtUGE2 fusion protein was first assembled in a gateway pDON-P3P2 vector (Life Sciences) to generate pDON-NST1-2A-AtUGE2-L3L2 plasmid. pDON-NST1-2A-AtUGE2-L3L2 vector was built using In-Fusion HD Cloning System (Clontech) to assemble PCR products of *At*UGE2 and pDON-L3L2 vector containing the encoding sequence of NST1-2A (synthesized by GenScript, Piscatway, NJ) such that the encoding sequence of *At*UGE2 was inserted in frame with the 3′end of NST1-2A and at 5′end of the attL2 sequences. *At*UGE2 and pDON-NST1-2A-L3L2 PCR products were generated using Phusion Polymerase with ntU2-F/ntU2-R primer pair and F-pDON-attL2/R-pDON-NST1-2A primer pair respectively. The NST1-2A-AtUGE2 gene fusion was then transferred from into pTKan-pIRX5-GWR3R2 by LR recombination (Life technologies) to express NST1-2A-AtUGE2 under the control of the fiber-specific pIRX5 promoter. *Pt*UGEc cDNA was generated from RNA isolated from Poplar leaf tissue. RNA was extracted using the RNeasy Plant Minikit (Qiagen), treated with DNase I (Sigma) and cDNA was generated using iScript™ Reverse Transcription Supermix (Biorad). The cloned UGE open reading frames were confirmed by sequencing to be identical to the published sequences (see Figure 1 for accession numbers). For stable transformation of *A. thaliana* wild-type Columbia-0, *uge4*/*rhd1-1* mutants or GalS1 overexpressing lines, constructs were transformed into *A. tumefaciens* strain C58-1 pGV3850 and plants were transformed using the floral dip method [22] (Table 2). Transformants were selected on MS medium containing hygromycin and transferred to soil. Plants confirmed to express the transgene were propagated and further characterized in the T2 and T3 generations.

**Table 1:** **Primer sequences used in this work**

**Primer name**	**Primer sequence**
fU2-F	5′-CACCATGGATTACAAGGATGACGATGACAAGGCGAAGAGTG-3′
fU2-R	5′-TTATGAAGAGGAGCCATTGGAGGAGGA-3′
fU4-F	5′-CACCATGGATTACAAGGATGACGATGACAAGGTTGGGAATATT-3′
fU4-R	5′-TTATGTTGAGTTTGGTGAAGAACCGTAACC-3′
fU5-F	5′-CACCATGGATTACAAGGATGACGATGACAAGATGGCTAGAAACGT-3′
fU5-R	5′-TTTAATGAGAGTTGTCTTCAGAAGAGG-3′
fUc-F	5′-CACCATGGATTACAAGGATGACGATGACAAGATGGCCTATAATATTC TGGTTACCG-3′
fUc-R	5′-TCAGTTTGTGCCGTCAGGAGATC-3′
ntU2-F	5′-TCTAATCCAGGACCTATGGCGAAGAGTGTTTTGGTTAC-3′
ntU2-R	5′-CAAGAAAGCTGGGTCTGAAGAGGAGCCATTGGAGGAG-3′
F-pDON-attL2	5′-GACCCAGCTTTCTTGTACAAAGT-3′
R-pDON-NST1-2A	5′-AGGTCCTGGATTAGACTCAACG-3′

**Table 2:** **Constructs used for plant expression**

**Name**	**Construct**	**Expression vector**	**Plant background**
*p35S:UGE2*	*p35S:FLAG-AtUGE2*	pMDC32	Col-0, *uge4,* GalS1-OE
*p35S:UGE4*	*p35S:FLAG-AtUGE4*	pMDC32	Col-0, *uge4*
*p35S:UGE5*	*p35S:FLAG-AtUGE5*	pMDC32	Col-0, *uge4*
*p35S:UGEc*	*p35S:FLAG-PtUGEc*	pMDC32	Col-0, *uge4*
*p35S:GalS1*	*p35S:YFP-GalS1*	pEarleyGate104	Col-0
*pIRX5:NST1-UGE2*	*pIRX5:NST1-2A-AtUGE2*	pTKan-pIRX5-GWR3R2	Col-0, GalS1-OE
EVC	*35S:pvu2* (non coding)	pMDC32	Col-0, *uge4*

### Plant growth conditions and measurements

All plants were grown in a 14–16 h photoperiod at 120 μmol m^−2^ s^−1^ photon flux density. Plants for root length measurements were grown vertically on MS plates and scanned at 6 days after germination. The root length of about 50 individual seedlings was measured using ImageJ [23].

### Monosaccharide composition analysis of cell walls

Bottom sections (5 cm length of stems) or whole leaves from 5-week-old plants were ground in liquid nitrogen and alcohol-insoluble residue (AIR) was prepared and enzymatically destarched as described [24]. Destarched AIR samples (1 mg) were hydrolyzed with 2 M triflouroacetic acid (TFA) and monosaccharide composition was determined by HPAEC-PAD as described [25,26].

### Expression and purification of His-*Pt*UGEc

*Pt*UGEc was introduced into pDEST17 expression vector (Invitrogen) containing an N-terminal 6xHis-tag and an IPTG inducible promoter. Gene expression in BL21 Star cells (Invitrogen) was induced by adding IPTG to a final concentration of 1 mM, and cultures were grown at 18°C overnight. *Pt*UGEc was purified from the supernatant of lysed cell pellets using HIS-Select Nickel Affinity Gel purification (Sigma-Aldrich). Lysis of bacterial cells took place using CelLytic B 2X containing 0.2 mg/ml lysozyme, 50 U/ml benzonase (all Sigma-Aldrich) and proteinase inhibitor cocktail (Roche). HIS-tagged *Pt*UGEc was desalted with PD-10 desalting columns (Amersham Biosciences). Samples of 30 μg His-*Pt*UGEc protein were separated on a Novex 8-16% Tris Glycine Gradient gel (Invitrogen) and stained with Coomassie Brilliant Blue.

### His-*Pt*UGEc activity assay

5 μg of purified His-*Pt*UGEc was mixed with 1 mM UDP-glucose in 50 mM Tris–HCl (pH 8.0), 1 mM DTT for 30 min at 30°C. Reactions were terminated by incubating for 10 min at 90°C and filtration through 0.45 μm filters (Millipore) prior to quantification. To test more nucleotide sugar substrates, the same reaction mixture as described above was used, substituting UDP-Glc with UDP-Gal, UDP-Xyl or UDP-Ara. Negative controls of purified protein boiled for 10 min at 90°C were used for all reactions. Separation and quantification of UDP-sugars in terminated reactions was performed by HPAEC analysis using a Dionex Ultimate 3000 system (Thermo Fisher) with detection at 262 nm. Samples were separated on a CarboPac PA20 column (Thermo Fisher) and eluted with an ammonium formate gradient according to Rautengarten *et al*. [27]. Standard solutions containing UDP-Glc, UDP-Gal (both Sigma Aldrich), UDP-Ara and UDP-Xyl (both Carbosource Service) were run as references.

### Immunofluorence microscopy

The top and base 3 cm of main stems from 6-week-old plants (3 stems/line) were harvested and fixed overnight at 4°C in fixative solution (4% paraformaldehyde in 50 mM piperazine-N-N’-bis(2-ethanesulphonic acid), 5 mM EGTA, pH 6.9). Fixed stem sections were embedded in 7% agarose and sectioned using a Leica VT1000S vibratome. Stem sections were labeled with monoclonal LM5 rabbit antibody (PlantProbes, Leeds, UK), which recognizes 1,4-linked β-galactan [28]. The labeling was performed according to Verhertbruggen et al. [29]. Sections were mounted on slides and pictures were taken using a LSM 710 confocal Microscope (Carl Zeiss). Lignin autofluorescence was monitored using a 405 nm Diode laser. Images were acquired with the Zen software package (Carl Zeiss) and analyzed with ImageJ [23].

## Abbreviations

GalA: α-D-galacturonic acid
GlcA: α-D-glucuronic acid
GalS1: GALACTAN SYNTHASE1
GT: Glycosyltransferase
NST: NAC secondary wall thickening promoting factor
NAC: NAM ATAF1/2 and CUC2
UGE: UDP-glucose 4-epimerase
UDP: Uridine diphosphate
